# Bavachin Rejuvenates Sensitivity of Colistin against Colistin-Resistant Gram-Negative Bacteria

**DOI:** 10.3390/ijms25042349

**Published:** 2024-02-16

**Authors:** Jie Li, Ning Han, Zhengyuan He, Xiaolan Dai, Feifei Zhao, Yangyang Li, Wenguang Xiong, Zhenling Zeng

**Affiliations:** 1 Guangdong Provincial Key Laboratory of Veterinary Pharmaceutics Development and Safety Evaluation, College of Veterinary Medicine, South China Agricultural University, Guangzhou 510642, China; xjzxlj@126.com (J.L.);; 2 National Risk Assessment Laboratory for Antimicrobial Resistance of Animal Original Bacteria, South China Agricultural University, Guangzhou 510642, Chinaxiongwg@scau.edu.cn (W.X.); 3 National Laboratory of Safety Evaluation (Environmental Assessment) of Veterinary Drugs, South China Agricultural University, Guangzhou 510642, China

**Keywords:** antibiotic adjuvant, bavachin, colistin, synergy effect, Gram-negative bacteria

## Abstract

The emergence of plasmid-mediated colistin resistance threatens the efficacy of colistin as a last-resort antibiotic used to treat infection caused by Gram-negative bacteria (GNB). Given the shortage of new antibiotics, the discovery of adjuvants to existing antibiotics is a promising strategy to combat infections caused by multidrug-resistant (MDR) GNB. This study was designed to investigate the potential synergistic antibacterial activity of bavachin, a bioactive compound extracted from the Psoralea Fructus, combined with colistin against MDR GNB. Herein, the synergistic efficacy in vitro and the therapeutic efficacy of colistin combined with bavachin in vivo were evaluated. The synergistic mechanism was detected by fluorescent probe and the transcript levels of *mcr*-1. Bavachin combined with colistin showed an excellent synergistic activity against GNB, as the FICI ≤ 0.5. In contrast to colistin alone, combination therapy dramatically increased the survival rate of *Galleria mellonella* and mice in vivo. Moreover, the combination of bavachin and colistin significantly reduced the amount of bacterial biofilm formation, improved the membrane disruption of colistin and inhibited *mcr*-1 transcription. These findings show that bavachin is a potential adjuvant of colistin, which may provide a new strategy to combat colistin-resistant bacteria infection with lower doses of colistin.

## 1. Introduction

Antibiotic resistance is an increasing threat to global public health [1]. The spread of multidrug resistance, especially in Gram-negative bacteria (GNB), makes antibiotic treatment ineffective. Bacteria such as *Escherichia coli* (*E. coli*), *Salmonella typhimurium* (*S. typhimurium*), *Klebsiella pneumoniae* (*K. pneumoniae*), *Pseudomonas aeruginosa* and *Acinetobacter baumannii* have exhibited extensive drug resistance to most available antibiotics, including the life-saving polymyxin and carbapenems [2]. It is reported that more than 700,000 people die from multidrug-resistant bacteria infection every year in the world, it may increase to 10 million by 2050 without effective control [3]. Alarmingly, the spread of the plasmid-borne colistin-resistant *mcr* gene in GNB has emerged worldwide, which leaves clinicians with few choices among the existing antibiotics [4,5]. Moreover, colistin is limited in clinical dosage and long-term therapy due to the nephrotoxicity and neurotoxicity [6]. Thus, alternative strategies are critically needed to tackle such infections. Drug combinations have emerged as a promising alternative approach to provide novel therapeutic options for multidrug-resistant bacteria [7]. Compared to developing new drugs, combination therapy with existing antibiotics could be more economical and faster [8,9].

At present, as the number of MDR bacteria continues to increase, the significance of natural products and Chinese herbal ingredients in bridging the gap in the development of new antibiotics or antibiotic alternatives cannot be overstated [10,11,12]. Most of these agents are plant-derived compounds, which are currently receiving more and more attention due to their rich structure and diverse sources [13,14,15]. For example, α-mangostin, a xanthone derivative from the pericarp of *Garcinia mangostana* L. peel extract, exhibits rapid bactericidal activity against Gram-positive bacteria and enhances the antibacterial activity of colistin against GNB [16,17]. A natural compound naringenin microsphere as the adjuvant reverses colistin resistance against MDR *K. pneumoniae* infection by various strategies [18]. Thymol, an essential oil from medicinal plants, potentiates the antibacterial activity of colistin by accelerating the damage and permeability of the bacterial outer membrane [19].

Bavachin, a member of the flavonoid family, is a compound extracted from the traditional herbal medicine Psoraleae Fructus [20]. As an important Chinese herbal medicine, Psoraleae Fructus is often used to treat osteoporosis and vitiligo [21]. At present, bavachin has been reported to be involved in multiple processes, including obesity suppression [20], anti-inflammation [22], anticancer [23], antibacterial [24], and immunoadjuvant activity [25]. In addition, it has been reported that bavachin exerted good antibacterial activity against Gram-positive bacteria by disrupting the cytoplasmic membrane (CM), whereas it has no antibacterial effect on GNB [24]. However, there has been no study on the synergistic activity between bavachin and colistin against GNB. Here, we found that bavachin could potentiate the sensitivity of colistin against GNB both in vitro and in vivo, providing a new potential treatment strategy for the treatment of colistin-resistant bacteria infection in the future. 

## 2. Results

### 2.1. Bavachin Combined with Colistin Has Strong Synergistic Effects on MDR GNB

**Figure 1:**
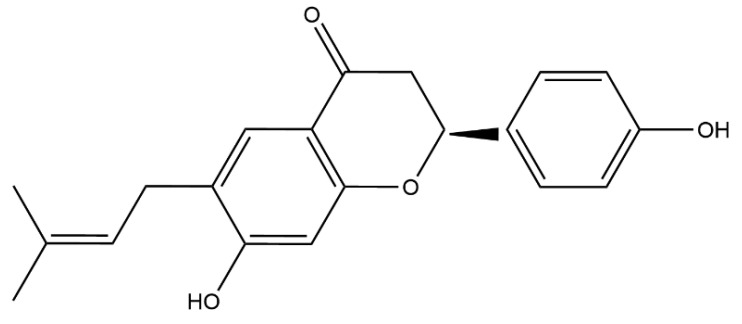
The chemical structure of bavachin.

**Figure 2:**
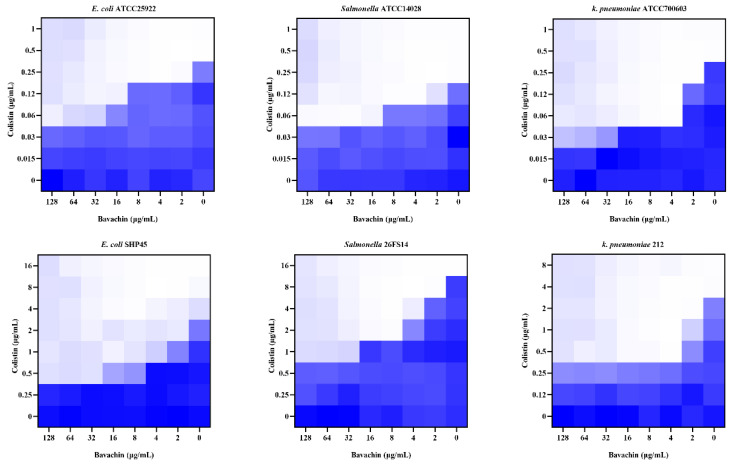
The synergistic effect of bavachin combined with colistin against colistin-resistant and -susceptible bacteria using the checkerboard assay. The data represent the values at OD_600_ nm of bacterial culture. Dark blue regions represent higher cell density.

To determine the synergistic activity of bavachin (Figure 1) and colistin, the MIC for bavachin was determined first. As shown in Table S1, bavachin alone has no antibacterial activity against GNB. Subsequently, we investigated the potential synergistic effect of bavachin in combination with colistin. The MIC values of colistin were reduced 4 to 8 fold when combined with bavachin (Figure 2), including *E. coli* ATCC 25922 (FICI = 0.125), *E. coli* SHP45 (FICI = 0.125), *K. pneumoniae* ATCC 700603 (FICI = 0.25), *K. pneumoniae* 212 (FICI = 0.125), *S. typhimurium* ATCC 14028 (FICI = 0.25), and *S. typhimurium* 26FS14 (FICI = 0.125). These results indicated that the synergistic antibacterial effect of bavachin combined with colistin was universal to the tested strains. In addition, compared with colistin alone, there was a negligible increase in the hemolytic activity of red blood cells with the combination treatment (Figure S1). These results suggested that bavachin was a potential adjunct of colistin without obvious toxicity.

### 2.2. Effect of the Colistin Combined with Bavachin on Antibacterial Activity and Bacterial Biofilm Formation

**Figure 3:**
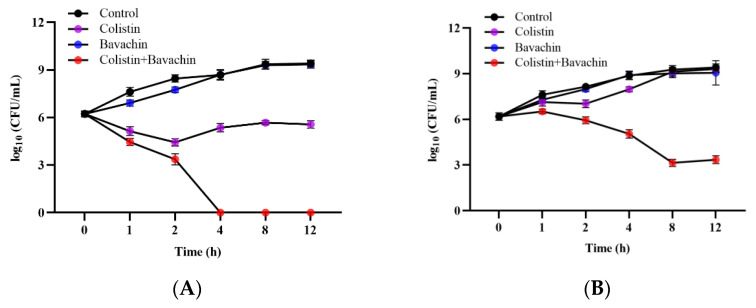
Potential antibacterial activity of colistin combined with bavachin against *E. coli* in vitro. (**A**) Time–kill curves of *E. coli* ATCC 25922 incubated with colistin (1 × MIC), bavachin (32 μg/mL) alone or their combination (0.5 × MIC colistin + 32 μg/mL bavachin). (**B**) Time–kill curves of *E. coli* SHP45 after exposing to 1 × MIC colistin, 32 μg/mL bavachin alone or their combination (0.5 × MIC colistin + 32 μg/mL bavachin). The initial density of the bacteria was approximately 10^6^ CFU/mL. All experiments were repeated three times independently, and experimental data are expressed as the mean ± SD.

To further evaluate the synergistic activity of colistin and bavachin in vitro, the time–kill assay was performed on *mcr*-positive bacteria (*E. coli* SHP45) and *mcr*-negative bacteria (*E. coli* ATCC 25922). After treatment with 32 μg/mL bavachin monotherapy, there was no effect on bacterial growth compared with the control. Using 1 × MIC of colistin alone resulted in a slight reduction in bacterial counts, while the antibacterial activity of colistin was enhanced in the presence of bavachin. Bacterial counts were rapidly reduced within 4 h when treated with the combination of the 0.5 × MIC colistin and 32 μg/mL bavachin (Figure 3). In particular, the bacterial number of *E. coli* ATCC 25922 was eradicated after treatment with 0.5 × MIC colistin in combination with 32 μg/mL bavachin (Figure 3A). These results showed that bavachin was a potent adjuvant to enhance the antimicrobial activity of colistin. 

**Figure 4:**
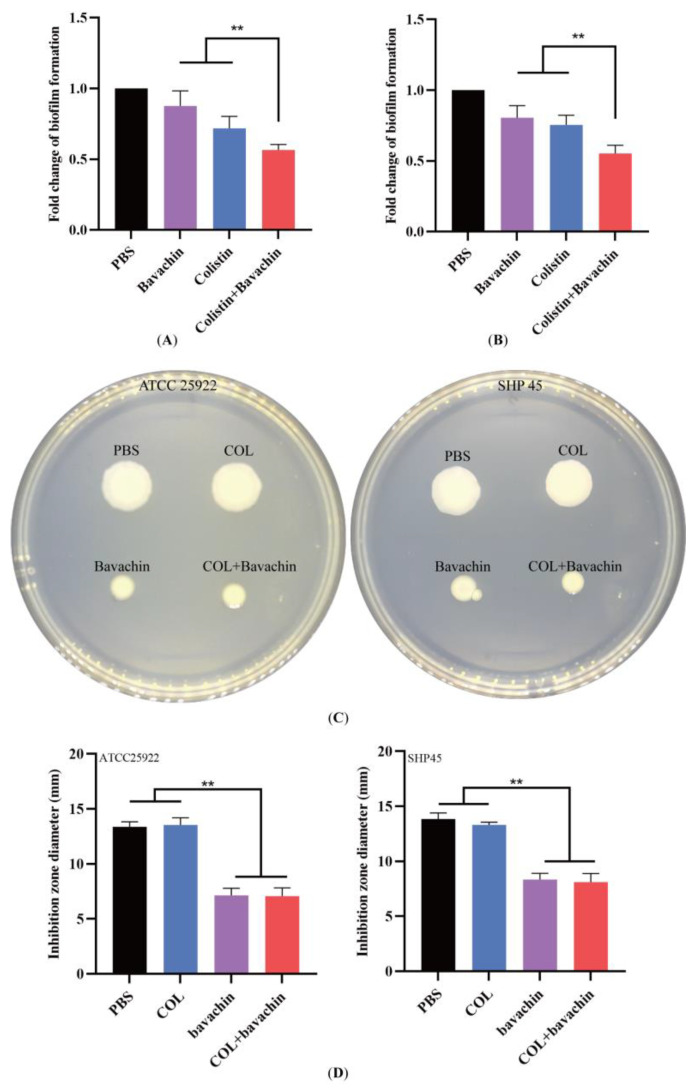
Effect of the colistin combined with bavachin on biofilm formation and swimming motility. (**A**) The biofilm formation of *E. coli* ATCC 25922 after exposure to the combination of colistin and bavachin (32 μg/mL bavachin + 0.5 × MIC colistin). (**B**) The biofilm formation of the combination of 0.5 × MIC colistin and 32 μg/mL bavachin against *E. coli* SHP45. (**C**) Bavachin inhibited the bacterial motility of *E. coli* without inhibition of bacterial growth. The bacteria were grown in 0.6% agar plates at 30 °C. (**D**) The diameter of the inhibition zone of 0.5 × MIC colistin combined with 32 μg/mL bavachin against *E. coli*. COL, colistin alone. COL + bavachin, the combination of colistin and bavachin. Data are representative of three independent experiments and expressed as the mean ± SD. *p* values are determined by nonparametric one-way ANOVA (** *p* < 0.01).

Biofilms are clusters of microbial cells that adhere to the outside of the cell and protect bacteria from antibiotic stress, and the formation of bacterial biofilm is thought to be one of the major factors contributing to the development of antibiotic resistance [26,27,28]. Therefore, the effect of colistin combined with bavachin on biofilms was investigated by means of crystal violet staining. In contrast to the colistin alone, the combination of 32 μg/mL bavachin and 0.5 × MIC colistin significantly reduced the amount of bacterial biofilm formation (Figure 4A,B). Bacterial biofilm formation is associated with motility [29,30], and we further evaluated whether the colistin combined with bavachin could inhibit bacterial motility using the semi-solid assay. As expected, the colistin in the presence of bavachin considerably suppressed the motility of the bacteria, whereas colistin alone had no effect on bacterial motility (Figure 4C). The results demonstrated that bavachin combined with colistin not only increased antibacterial activity of colistin, but also inhibited bacterial biofilm formation and motility.

### 2.3. Bavachin Enhances the Efficacy of Colistin In Vivo Infection Models

**Figure 5:**
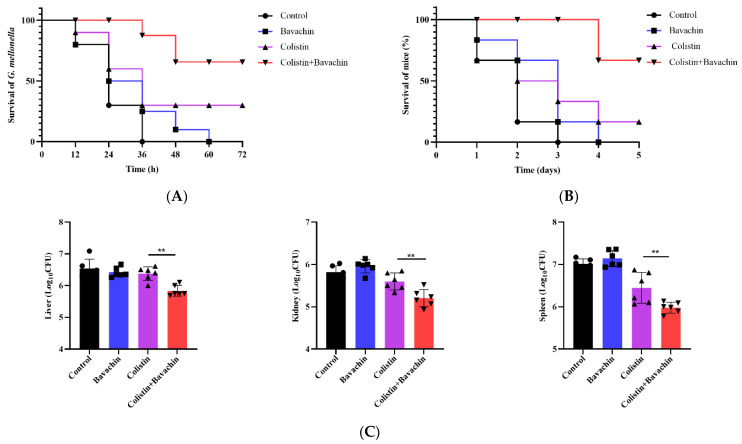
Bavachin enhances the activity of colistin in animal infection models. (**A**) The survival rates of *Galleria mellonella* larva (*n* = 10 per group) with different treatments post infection. The *Galleria mellonella* larva are infected with colistin-resistant *E. coli* SHP45 and treated with 4 mg/kg colistin, 32 mg/kg bavachin alone, or their combination (2 mg/kg colistin + 32 mg/kg bavachin). (**B**) The survival rates of mice (*n* = 6 per group) in the mouse infection model. The survival rates of mice are increased after treatment with the combination of colistin and bavachin (2 mg/kg + 32 mg/kg). (**C**) Bacterial loads of different organs in the mouse peritonitis–sepsis model. Organs included liver, kidney and spleen. All data are presented as the mean ± SD from three independent experiments. The significances are determined by nonparametric one-way ANOVA (** *p* < 0.01).

Given that the combination of colistin and bavachin displayed outstanding synergistic bactericidal activity against GNB in vitro, we next investigated the therapeutic efficacy in vivo using two animal infection models. Firstly, the survival rates of *Galleria mellonella* larvae infected by *E. coli* SHP45 were recorded at various time points. All larvae treated with PBS died within 36 h after treatment. The survival rate of larvae treated with bavachin monotherapy was similar to the PBS group, and all died within 60 h after treatment. Approximately 30% of infected larvae survived after treatment with 4 mg/kg colistin, while the survival rate treated of colistin combined with bavachin (2 mg/kg + 32 mg/kg) could reach 70% (Figure 5A). In the mouse sepsis model, the survival rates and organ burden of mice were used to evaluate the effect of synergistic therapy. All mice treated with PBS or bavachin died within 4 d. Similarly, compared to colistin monotherapy, the combination of 2 mg/kg colistin and 32 mg/kg bavachin improved survival from 16.6% to 83.3% (Figure 5B). In addition, the bacterial burden in various organs, spleen, liver, and kidney included, was reduced in the combination therapy group (Figure 5C). These results indicated that bavachin could improve the therapeutic activity of colistin in vivo against emerging resistant bacteria.

### 2.4. Bavachin Plays an Important Role in the Function of the MCR Protein

**Figure 6:**
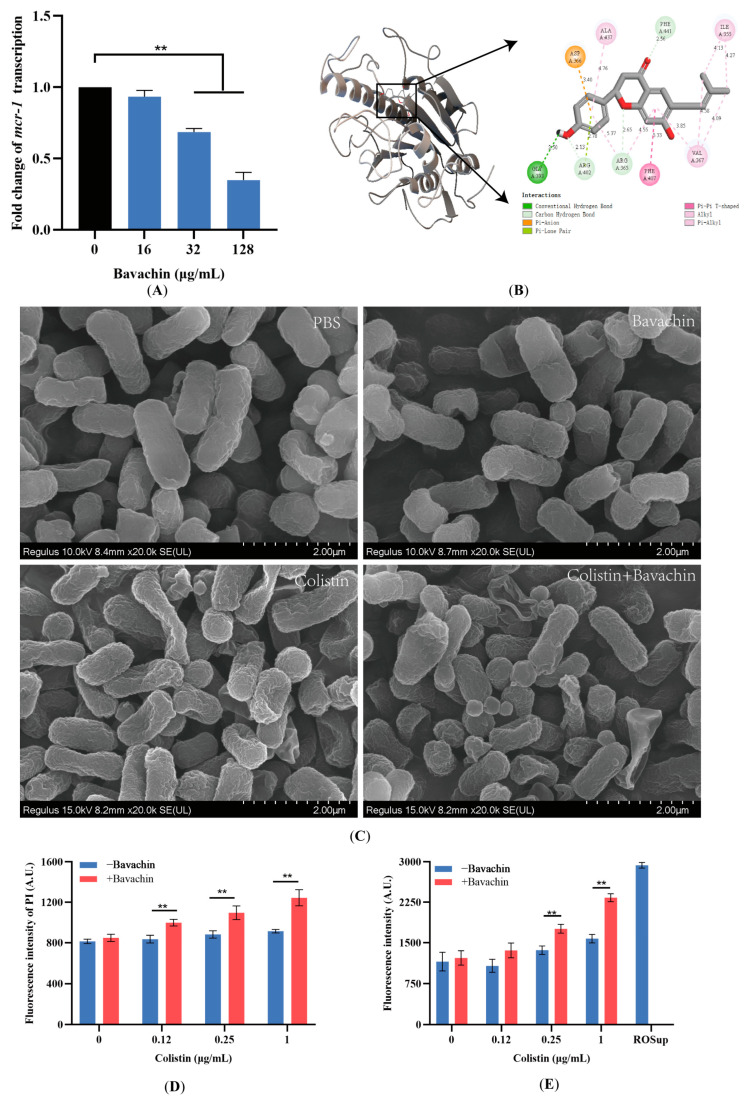
Bavachin enhances the bacterial membrane disruption ability of colistin. (**A**) Relative transcript levels of *mcr*-1 after treatment with different concentrations of bavachin. (**B**) Potential binding mode between bavachin and MCR-1 protein using molecular docking. The left panel is the MCR-bavachin complex; the right panel is the 2D structure of the interaction between MCR-1 and bavachin. (**C**) Morphological analysis of *E. coli* ATCC 25922 after exposure to 0.5 × MIC colistin, 32 μg/mL bavachin alone, or their combination (0.5 × MIC colistin + 32 μg/mL bavachin) using scanning electron microscopy (scale bar: 2 μm). (**D**) Detection of the cytoplasmic membrane permeability of *E. coli* under different concentrations of colistin (0, 0.12, 0.25 and 1 μg/mL) with or without 32 μg/mL bavachin. (**E**) The accumulation of ROS of different concentrations of colistin (0, 0.12, 0.25 and 1 μg/mL) in the absence or presence of 32 μg/mL bavachin. Data are expressed as the mean ± SD from triplicate analyses. The significances are determined by multiple t tests, ** *p* < 0.01.

To further understand the synergistic mechanism of bavachin and colistin combination, the transcript levels of *mcr*-1 were detected by qRT-PCR. We found that the bavachin inhibited *mcr*-1 transcription in a dose-dependent manner (Figure 6A), indicating that bavachin was a potential inhibitor to *mcr*-1 transcription. Moreover, we predicted the potential binding sites of bavachin and MCR using molecular docking. Bavachin could bind to active amino acid residues in the pocket of MCR protein, including ALA437, PHE441, ARG365, PHE407, and GLY393 (Figure 6B and Figure S2A). Moreover, the binding energy was −6.51 kcal/mol, indicating that bavachin has a good affinity with MCR. These results showed that bavachin affected the function of the MCR protein by binding to amino acid residues, which may be one of the reasons why the combination of FFA and colistin has a synergistic effect on mcr-positive strains.

### 2.5. Bavachin Potentiates the Membrane-Damaging Activity of Colistin 

Considering that bavachin could enhance the antibacterial activity of colistin against *mcr*-negative bacteria, more specific mechanisms still require exploration. Firstly, the morphology and structure of bacteria treated with different agents were observed by SEM. Compared with the control group, bacterial morphology was slightly affected in the both the colistin and bavachin monotherapy groups. However, the surface of the bacteria in the colistin and bavachin combination group displayed indentations and dissolution, which caused obvious damage to the integrity of the cell membrane structure (Figure 6C). The fluorescent probe PI was used to further assess CM permeability, and bavachin alone has no effect on the CM. In contrast to colistin alone, the fluorescence levels of PI were significantly increased when combined with bavachin (*p* < 0.05) (Figure 6D), indicating that bavachin alone has no effect on bacterial membrane. Interestingly, there was no significant increase in intracellular protein leakage (*p* > 0.05) (Figure S2B,C), indicating that the structural damage caused by the combination of bavachin and colistin to the bacterial membrane is not sufficient to cause random leakage of macromolecular substances such as proteins. Disruption of the CM often leads to the accumulation of ROS. Therefore, the levels of ROS were measured after treatment with the combination of colistin and bavachin. Compared with colistin alone, we found that ROS levels were significantly increased in the presence of bavachin (*p* < 0.05) (Figure 6E). These results indicated that the combination of bavachin and colistin could disrupt the bacterial membrane structure, increase the permeability of the CM and promote the accumulation of ROS.

## 3. Discussion

The continued increase in bacterial resistance poses a huge threat to public health and is a matter of public concern [31]. Infection with MDR bacteria severely threatens the therapeutic efficacy of the last-resort antibiotic and limits the choice of clinical drugs [32]. Compared to the high cost and long cycle time of developing new drugs, discovering antibiotic synergists of existing antibiotics is a promising strategy to combat antibiotic-resistant bacteria [33]. In this study, a new colistin potential adjuvant, bavachin, was identified. We investigated the potential synergistic antibacterial effect of bavachin combined with colistin, and the results showed that bavachin was able to enhance the antibacterial efficacy of colistin. Moreover, bavachin could potentiate the therapeutic effect of colistin in mice and *Galleria mellonella* larvae animal models in vivo. This finding provides a potential option to combat the infection caused by colistin-resistant strains.

Flavonoids are widespread in plants [34,35], and there are currently more than 6000 structures known [36]. Flavonoids are attracting more and more attention in nutrients, health supplements [37], human health [38], tumor therapy [39] and anti-inflammatory activity [40]. However, despite so many effects, safety is an important indicator for clinical application in future. We found a slight increase in the hemolytic capacity of sheep red blood cells with the combination of colistin and bavachin. Furthermore, the toxicity of bavachin was measured on A549 cells, and it has been reported that bavachin has a lower toxic effect at low concentrations [41]. In addition, the combination of bavachin and colistin could reduce the dosage of colistin, thereby reducing the toxic side effects of colistin. Therefore, bavachin is a potential adjuvant for colistin. 

In recent years, there have been some reports on the combination of colistin and nonantibacterial agents [42,43]. However, compared with chemically synthesized small molecules, traditional Chinese medicine has unique physicochemical properties and a wide range of pharmacological activities. Thus, bavachin may play an important role in the combination of bavachin and colistin. Biofilm is the three-dimensional community of bacteria [44], made up of extracellular polymeric substances, which is a mixture of polysaccharide, protein, extracellular DNA, and others [26]. The formation of biofilm can protect bacteria from the disturbance of the external environment, leading to an increase in persistent bacterial infection and increased antibiotic resistance [45]. Therefore, antibiofilm activity is also a critical requirement in the screening of new antibiotics or antibiotic adjuvants. In this study, bavachin combined with colistin can effectively inhibit the formation of biofilms. Furthermore, we used molecular docking to explore the potential binding modes of bavachin and MCR, while functional verification of this binding mode and further experiments on the MCR protein are still needed. 

Membrane permeability is one of the important factors for antibiotic activity and membrane-targeted antibiotics usually have fast bactericidal activity [3,19,46]. Bavachin has been reported to destroy the CM of Gram-positive bacteria in a dose-dependent manner [24]. In this study, we found that bavachin alone had no effect on CM of Gram-negative bacteria, it is possible that the low permeability of the outer membrane prevents agents from entering the cell [47]. However, the addition of bavachin can increase the ability of colistin to destroy the bacterial membrane. Moreover, the combination of bavachin and colistin showed a rapid bactericidal effect in the time–killing curve, the bacterial count dropped rapidly within 4 h. The integrity of the bacterial membrane structure is the prerequisite for the normal operation of various biological functions in the bacteria [48]. Thus, the combination of bavachin and colistin exerts a synergistic antibacterial effect by disrupting the membrane structure.

Previous studies reported that bavachin was rapidly absorbed into the plasma of rats 1 h after oral administration and evenly distributed into the brain nuclei (t_max_ = 1 h) [49]. In addition, studies showed that the oral bioavailability of bavachin is significantly affected by intestinal and renal oxidation and glucuronidation [50]. However, drug–drug interactions may increase clinical drug plasma concentrations in clinic. Therefore, combination with bavachin is also a potential approach to overcome limitations.

## 4. Materials and Methods

### 4.1. Reagents and Bacterial Strains 

Bavachin (analytical standard, CAS No. 19879-32-4), colistin sulfate (CAS No. 1264-72-8) and tigecycline (CAS No. 220620-09-7) were purchased from Macklin Biochemical Co., Ltd. (Shanghai, China), Sigma-Aldrich (St. Louis, MO, USA), and Dalian Meilun Biotech Co., Ltd. (Dalian, China), respectively. All other antibiotics were from Sangon Biotech Co., Ltd. (Shanghai, China). All strains used in this study are listed in Table S1. The clinical isolates of *E. coli*, *S. typhimurium* and *K. pneumoniae* were isolated and preserved in our laboratory.

### 4.2. The Antimicrobial Susceptibility Test

The minimum inhibitory concentration (MIC) values of antibiotics were determined by the broth microdilution method according to the Clinical and Laboratory Standards Institute (CLSI) [51]. Bacteria were cultured to the logarithmic growth phase, and adjusted to a cell density of 10^6^ CFU. Antibiotics were 2-fold serial dilutions in the Mueller–Hinton (MH) broth. The MIC was recorded with no visible growth after incubation at 37 °C for 16 h. Synergistic antibacterial activity was determined by calculating the fractional inhibitory concentration index (FICI) through the checkerboard experiment. A FICI ≤ 0.5 was deemed as synergistic, a 0.5 ≤ FICI ≤ 1 was deemed as addition, a 1 < FICI ≤ 4 was deemed as indifference and a FICI > 4 was defined as antagonism.

### 4.3. The Hemolytic Activity Assay

The hemolytic activity of colistin with or without bavachin was determined with sheep blood cells according to a previous study [52]. Sterile sheep blood was washed twice, and 8% red cells was exposed to varying concentrations of colistin (1, 2, 4 and 16 μg/mL) in the absence or presence of bavachin (32 μg/mL). Meanwhile, Triton X-100 and phosphate-buffered saline (PBS) were used as a positive control and a negative control, respectively. After 1 h, the supernatant was centrifuged and the absorbance was measured at 576 nm. The calculation of the hemolysis rate is as follows:

$$Hemolysis\;rate\left(\%\right)=\frac{\left({OD}_{sample}-{OD}_{PBS}\right)}{\left({OD}_{TritonX-100}-{OD}_{PBS}\right)}\times 100\%$$

### 4.4. The Time–Kill Assay

The antibacterial activity of the single agent or the combination of the colistin and bavachin was determined by time–kill curve. Briefly, bacteria in the exponential phase were diluted to approximately 10^6^ CFUs, and the bacterial suspension was treated with 1 × MIC colistin alone, 32 μg/mL bavachin alone or their combination (0.5 × MIC colistin + 32 μg/mL bavachin). At the same time, the control group was treated with PBS. The changes in bacterial count were measured over 12 h by plating serial samples. After overnight cultivation at 37 °C, the colony counts were counted on the agar plates. The experiment was repeated at least three times.

### 4.5. The Biofilm Formation Assay

Bacterial biofilm formation was measured by crystal violet staining based on a previous, slightly modified method in [53]. Briefly, bacterial cultures of *E. coli* were diluted to 10^6^ CFU, and then PBS, 0.5 × MIC colistin alone, 32 μg/mL bavachin alone or their combination (0.5 × MIC colistin + 32 μg/mL bavachin) was used to treat *E. coli* for 24 h at 37 °C in a 96-well flat bottom plate. Eight wells were used for each agent concentration. Subsequently, the supernatant was discarded and planktonic bacteria were washed with PBS. The biofilms were fixed with methanol and stained with 1% crystal violet. Finally, the biofilm was solubilized with 200 μL of 95% ethanol and the OD value was measured at 570 nm.

### 4.6. Motility Assay

Overnight-cultured *E. coli* was inoculated into fresh LB medium, and then incubated with PBS, 32 μg/mL bavachin, 0.5 × MIC colistin or their combination (32 μg/mL bavachin + 0.5 × MIC colistin). After 4 h, 2 μL of the bacterial culture from different groups were spotted onto semi-solid agar. After overnight incubation at 30 °C, photos were taken and the diameters of bacterial motility were measured.

### 4.7. RNA Isolation and Reverse Transcription (RT)-PCR

*E. coli* SHP45 was grown overnight and incubated with various concentrations of bavachin (0, 16, 32 and 128 μg/mL) for 4 h. The bacterial pellet was collected by centrifugation and total RNA was extracted by the FastPure Cell/Tissue Total RNA Isolation Kit V2 (Vazyme, Nanjing, China) according to the manufacturer’s instructions. cDNA was generated using the PrimeScript™ 1st Strand cDNA Synthesis Kit (Takara, Beijing, China). 16S rRNA was used as the housekeeping gene, and the relative expression of *mcr*-1 was calculated using the 2^−ΔΔCt^ method. Three independent experiments were performed with three replicate wells each. The sequences of primer are listed in Table S2.

### 4.8. The Molecular Docking Assay

A comparative assessment of the potential binding capacity of bavachin and MCR protein was performed using molecular docking. The structure of the MCR-1 protein was obtained from the RCSB protein crystals database (PDB entry: 5GRR) [18]. The structure of bavachin was drawn using ChemDraw 16.0 software. Before molecular docking, proteins were dehydrated, hydrogenated, and energy-optimized, and metal ions were removed. Molecular docking was performed in a grid box covering the entire protein molecule using the blind docking mode via AutoDock Vina 1.1.2 software. The binding energy for the target protein ligand was obtained using the Autodock Vina and the docking patterns was showed by Discovery Studio 2019 client.

### 4.9. Scanning Electron Microscopy (SEM)

The morphological appearance and morphometric analysis of the cell membrane of *E. coli* ATCC 25922 were determined using SEM. Briefly, the bacterial suspensions were washed twice with PBS and exposed to 32 μg/mL bavachin, 0.12 μg/mL colistin alone or their combination (32 μg/mL bavachin + 0.12 μg/mL colistin) at 37 °C. After 4 h, the cells were centrifuged, collected and fixed overnight using 2.5% glutaraldehyde at 4 °C overnight. Micrographs of the cells were taken using a super-resolution field-emission SEM (Hitachi, Tokyo, Japan).

### 4.10. The Protein Leakage Assay

The amount of protein leakage was detected by using a BCA detection kit. Bacteria were resuspended in PBS to an OD_600_ of approximately 0.5, and incubated with various concentrations of colistin with 32 μg/mL bavachin at 37 °C for 6 h. The suspension was centrifuged at 5000 rpm for 8 min and the supernatant was collected. A volume of 45 μL of sample and 200 μL of BCA working solution were added to each well, and the protein concentration was measured at a wavelength of 562 nm. Each sample was replicated with three wells, and the experiment was repeated three times.

### 4.11. Membrane Permeability Evaluation 

The permeability of the CM was assessed using the fluorescent probe propidium iodide (PI). Bacteria were washed twice and treated with different concentrations of colistin with or without 32 μg/mL bavachin for 15 min. Next, the PI fluorescent probe was added at a final concentration of 10 nM and the mixture was incubated for 20 min. The fluorescence intensity was measured with an excitation wavelength of 535 nm and an emission wavelength of 615 nm. All tests were performed in triplicate.

### 4.12. Reactive Oxygen Species (ROS) Measurement

The levels of ROS in *E. coli* were detected by fluorescent probe 2′,7′-dichloro fluorescein diacetate (DCFH-DA) with a final concentration of 10 μM. In brief, overnight cultures of *E. coli* ATCC 25922 were washed with PBS three times. The cultures were stained with DCFH-DA for 20 min in the dark. Then, the *E. coli* cells were treated with varied concentrations of colistin alone or combined with 32 μg/mL bavachin. After 1 h, the supernatant was collected by centrifugation and the fluorescence intensity of the DCFH-DA dye was determined at an excitation wavelength of 488 nm and an emission wavelength of 525 nm.

### 4.13. The Galleria Mellonella Infection Model

Initial assessment of treatment effects of colistin combined with bavachin using *Galleria mellonella* larvae in vivo was carried out. First, *Galleria mellonella* larvae were divided into four groups (10 larvae in each group) by block randomization, and infected with 1.6 × 10^5^ CFUs *E. coli* SHP45 suspension (10 μL) into the right proleg. After 1 h, 10 μL of PBS, colistin (4 mg/kg), bavachin alone (32 mg/kg) or the combination of colistin and bavachin (2 mg/kg + 32 mg/kg) was treated through left proleg. Then, the larvae were incubated at 37 °C and the number of larval deaths was counted within 72 h.

### 4.14. The Mice Infection Model In Vivo

Specified pathogen-free (SPF) female BALB/c mice (5 to 6 weeks old) were randomly grouped, with six mice in each group. Before the formal experiment, the mice were fed in an SPF environment for one week to adapt to the environment. *E. coli* SHP45 in the logarithmic growth phase were diluted to 1.6 × 10^8^ CFU, then 100 μL of bacterial suspension was administered by intraperitoneal injection. At 1 h post bacterial inoculation, four groups of mice were intraperitoneally injected with different agents, including PBS, colistin monotherapy (4 mg/kg), bavachin monotherapy (32 mg/kg) or their combination (2 mg/kg + 32 mg/kg). The mice were observed daily and the number of dead mice were recorded from the start of treatment. Once dead, the liver, spleen and kidney of mice were harvested aseptically. Mouse organs were homogenized and serially diluted, and then placed on the LB agar plates containing 2 μg/mL colistin for counting. After 5 d, all mice were euthanized by cervical dislocation and the bacterial colonies of organs were counted.

### 4.15. Statistical Analysis

The sample size for each statistical analysis was greater than or equal to three. All data were analyzed using GraphPad Prism 9.0 software. Comparison between 2 groups was calculated using the *t*-test. Comparison among 3 or more groups was performed using one-way ANOVA. * *p* < 0.05 and ** *p* < 0.01.

## 5. Conclusions

In conclusion, our data suggested that bavachin had a potential synergistic activity with colistin and inhibited bacterial biofilm formation. Furthermore, combination therapy can improve the therapeutic effect of colistin against MDR bacterial infections in vivo. Moreover, mechanistic studies showed that bavachin enhanced the ability of colistin to damage membrane and promoted the accumulation of ROS and intracellular colistin. However, more animal models in clinical practice and the pharmacokinetics of bavachin still need to be explored in future work. In general, facing the vacancy of new antibiotics, the discovery of bavachin as a new colistin adjuvant provides new ideas for the prevention and treatment of colistin-resistant bacteria.

## Data Availability

The original data and materials in this study were provided in this article/Supplementary Material, and further inquiries can be directed to the authors.

## Supplementary Materials

The following supporting information can be downloaded at: https://www.mdpi.com/article/10.3390/ijms25042349/s1.

## Abbreviations

*E. coli*	*Escherichia coli*
GNB	Gram-negative bacteria
MDR	Multidrug resistant
CM	Cytoplasmic membrane
CLSI	Clinical and laboratory standards institute
MH	Mueller–Hinton
SEM	Scanning electron microscopy
FICI	Fractional inhibitory concentration index
PBS	Phosphate-buffered saline
PI	Propidium iodide
SPF	Specified pathogen free
MIC	Minimum inhibitory concentration
*S. typhimurium*	*Salmonella typhimurium*
*K. pneumoniae*	*Klebsiella pneumoniae*
