# Supplementary material for: Bavachin Rejuvenates Sensitivity of Colistin against Colistin-Resistant Gram-Negative Bacteria
Source: Int J Mol Sci. 2024 Feb 16;25(4):2349. doi: 10.3390/ijms25042349 (PMC10889384; doi:10.3390/ijms25042349)
Supplement: Supplementary file 1 [file ijms-25-02349-s001.zip › ijms-2845338-supplementary.pdf]

Bavachin rejuvenates sensitivity of colistin against colistin-resistant Gram-negative bacteria

Jie Li [a, c], Ning Han [a, c], Zhengyuan He [a, b], Xiaolan Dai [a, b], Feifei Zhao [b, c], Yangyang Li [b], Wenguang Xiong [c], Zhenling Zeng [a, c] *

a Guangdong Provincial Key Laboratory of Veterinary Pharmaceutics Development and Safety Evaluation, College of Veterinary Medicine, South China Agricultural University, Guangzhou 510642, China.

b National Laboratory of Safety Evaluation (Environmental Assessment) of Veterinary Drugs, South China Agricultural University, Guangzhou 510642, China.

c National Risk Assessment Laboratory for Antimicrobial Resistance of Animal Original Bacteria, South China Agricultural University, Guangzhou 510642, China.

*Corresponding author: Zhenling Zeng

E-mail: zlzeng@scau.edu.cn

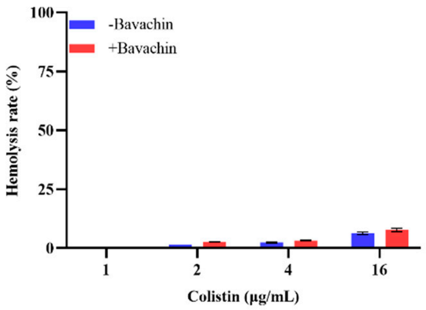

Figure S1 Hemolytic activity of colistin (1, 2, 4 and 16 μg/mL) in the absence or presence of 32 μg/mL bavachin. Triton X-100 and PBS were used as positive control and negative control, respectively.

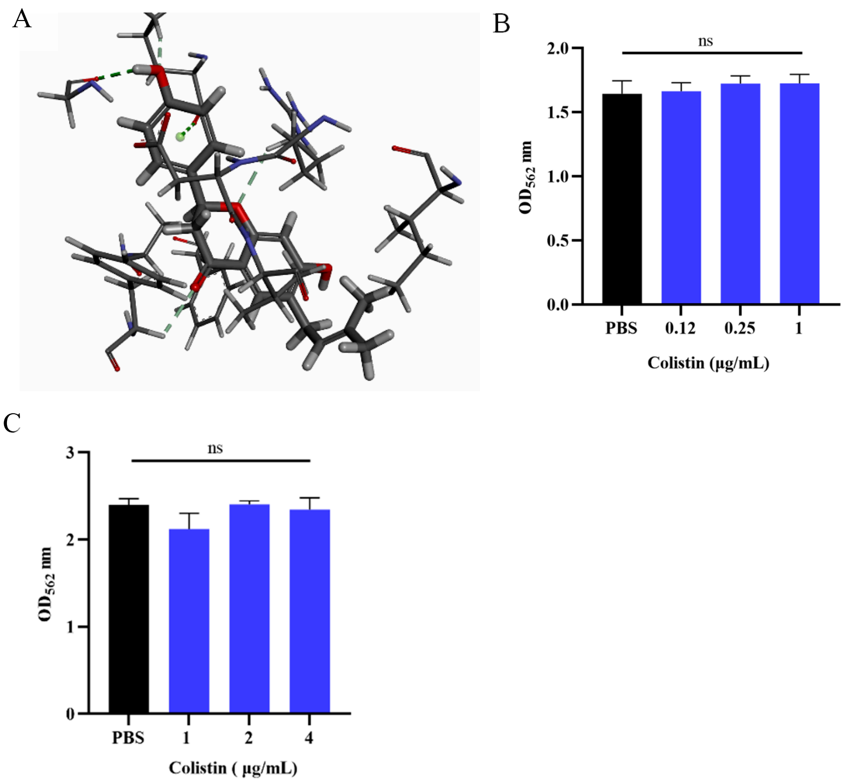

Figure S2 Synergistic antibacterial mechanism of bavachin combined with colistin against Gram-negative bacteria.

(A) The potential binding mode of bavachin and colistin by molecular docking.

(B) and (C) Bacterial protein leakage of *E. coli* ATCC 25922 (B) and *E. coli* SHP45 (C) after treatment with different concentrations of colistin in the present of 32 μg/mL bavachin for 6 h.

Table S1 The MIC values of bavachin and colistin against Gram-negative bacteria

**strain**	***mcr* gene**	**MIC (μg/mL)**		**FICI**
		colistin	bavachin	
E. coli ATCC25922	negative	0.25	> 128	0.125
E. coli SHP45	positive	4	> 128	0.125
E. coli GDQ8D147	negative	8	> 128	0.25
E. coli GDQ8P27	positive	4	> 128	0.25
P. multocida CVCC434	negative	0.5	> 128	0.125
K. pneumoniae 212	negative	4	> 128	0.125
K. pneumoniae ATCC700603	negative	0.5	> 128	0.25
S. typhimurium 26FS14	positive	2	> 128	0.125
S. typhimurium S226	positive	2	> 128	0.125
S. typhimurium ATCC14028	negative	0.25	> 128	0.25

Table S2 The qRT-PCR primer used in this study

Primer names	Sequence (5' to 3')	Size (bp)
mcr-1 -F	AAAGACGCGGTACAAGCAAC	213
mcr-1-R	GCTGAACATACACGGCACAG	
16s- F	TCCTACGGGAGGCAGCAGT	467
16s -R	GGACTACCAGGGTATCTAATCCTGTT	
